# Biochar application alleviates drought-induced oxidative stress by activating the salicylic acid-mediated glutathione synthesis pathway in *Brassica napus*

**DOI:** 10.1186/s12870-025-07575-7

**Published:** 2025-11-17

**Authors:** Bok-Rye Lee, Sang-Hyun Park, Muchamad Muchlas, Dong-Won Bae, Tae-Hwan Kim

**Affiliations:** 1 Department of Animal Science, College of Agriculture & Life Science, Chonnam National University, Gwangju, 61186 Republic of Korea; 2 Institute of Environmentally‑Friendly Agriculture (IEFA), Chonnam National University, Gwangju, 61186 Republic of Korea; 3 Faculty of Animal Science, University of Brawijaya, Veteran Street, Malang, East Java, 66245 Indonesia; 4 Core‑Facility Center for High‑Tech Materials Analysis, Gyeongsang National University, Jinju, 52828 Republic of Korea

**Keywords:** Biochar, Drought, Glutathione metabolism, Oxidative stress, Salicylic acid

## Abstract

Drought stress induces oxidative damage that disrupts cellular redox homeostasis. Biochar has recently attracted attention for its potential to enhance antioxidant defense systems and reduce reactive oxygen species (ROS) accumulation under drought conditions. This study aimed to investigate the mechanisms by which biochar alleviates drought-induced oxidative stress in *Brassica napus*, focusing on hormonal regulatory pathway in glutathione (GSH)-based redox control. The plants were grown under well-watered (Control), drought stress (Drought), or drought stress with biochar application (Drought + Biochar) conditions for 43 days. Drought increased ROS (O₂⁻and H₂O₂) and malondialdehyde levels, while reducing soil water content, shoot biomass, relative water content, and chlorophyll concentration. These changes were accompanied by increased abscisic acid (ABA) levels and the upregulation of ABA biosynthesis and signaling genes (*NCED3* and *ABI5*). Drought also decreased GSH content, GSH/GSSG ratio, *GSH1* expression, and GR activity, indicating severe oxidative stress and impaired redox homeostasis. Biochar application significantly alleviated drought-induced ROS accumulation and lipid peroxidation. Compared with drought alone, biochar promoted SA accumulation by 1.5-fold, upregulated the expression of SA biosynthesis (*ICS1*) and signaling (*NPR1*) by 5.6- and 3.3-fold, respectively, and reduced ABA content by 28%. Biochar also enhanced GSH levels (2.9-fold) and GSH/GSSG ratio (4.5-fold), accompanied by the upregulation of *GSH1* (4.2-fold) and suppression of *GPX7* (60.7%), relative to drought alone. Correlation analysis revealed a strong association between SA, GSH, and ROS. These findings indicate that biochar alleviates drought-induced oxidative stress by activating the SA-mediated GSH biosynthesis pathway and antagonizing ABA signaling, thereby enhancing antioxidant defense mechanisms and improving drought stress resilience in *Brassica napus*.

**Supplementary Information:**

The online version contains supplementary material available at 10.1186/s12870-025-07575-7.

## Introduction

Drought stress is a prevalent abiotic factor that significantly constrains plant growth and productivity. Under drought conditions, cellular homeostasis is disrupted, frequently resulting in the excessive accumulation of reactive oxygen species (ROS), including hydrogen peroxide (H₂O₂), superoxide anion (O₂⁻), hydroxyl radicals (•OH), and singlet oxygen (¹O₂). These ROS can damage cellular macromolecules, including proteins, lipids, and nucleic acids [1, 2]. To mitigate this oxidative stress, plants deploy a complex antioxidant defense system comprising both enzymatic components—such as superoxide dismutase (SOD), catalase (CAT), and ascorbate peroxidase (APX)—and non-enzymatic antioxidants such as ascorbate, glutathione (GSH), and flavonoids [3, 4]. Additionally, compatible solutes including glycine betaine and polyamines contribute to cellular protection by maintaining osmotic balance and supporting antioxidant defense systems under stress conditions [5, 6]. Among the various antioxidant components, GSH plays a central role in redox regulation by directly detoxifying ROS and maintaining cellular redox homeostasis [7]. GSH is synthesized through a two-step enzymatic process involving γ-glutamylcysteine synthetase (γ-ECS, encoded by GSH1) and glutathione synthetase (GSH2). Additionally, glutathione reductase (GR) regenerates reduced GSH from its oxidized form (GSSG), thereby sustaining the cellular GSH pool [7, 8].

Salicylic acid (SA), a phytohormone well-known for its role in plant immunity [9], is increasingly recognized as a key regulator of redox signaling under abiotic stresses, especially drought [10]. It enhances stress tolerance by upregulating both the expression and activity of key antioxidant enzymes, such as SOD, CAT, APX, GR, and dehydroascorbate reductase (DHAR) [11, 12]. This regulation facilitates ROS scavenging and helps maintain redox homeostasis during stress. SA enhances antioxidant defense by activating the ascorbate-glutathione (AsA-GSH) cycle, through which SA upregulates both the enzymatic activities and transcript levels of genes encoding GSH-related enzymes (e.g., *GR*, *GSH1*, and *GSH2*) [13, 14]. Furthermore, in our previous study, exogenous SA treatment increased antioxidant enzyme activity and stimulated the biosynthesis of reduced glutathione (GSH), thereby enhancing ROS scavenging under drought stress [15, 16]. In addition, drought-induced oxidative stress is exacerbated by ABA-dependent suppression of GSH biosynthesis and enhanced GSH oxidation, further disrupting redox homeostasis [17, 18]. Despite the increasing evidence of hormonal interaction with ROS in GSH synthesis pathway, hormonal regulatory pathway in GSH-based redox control has been still to be clearly elucidated.

Biochar, produced through the pyrolysis of organic matter, is a carbon-rich material that has been extensively studied for its beneficial effects on the physical and chemical properties of soils. It enhances soil porosity [19], increases water-holding capacity [20], and reduces bulk density [21]. Additionally, biochar modifies soil pH [22], improves nutrient availability [23], and increases the cation exchange capacity of the soil matrix [24]. Numerous studies have shown that biochar application improves drought tolerance by enhancing water relation, which positively modulates photosynthetic activity [25–27]. It also supports cellular hydration and preserves membrane integrity by reducing the accumulation of ROS [28–30] and alleviates lipid peroxidation [29, 30]. In previous studies, the positive effectiveness of biochar application in drought stress resistance have been mainly assessed by measuring the activity of antioxidant enzymes, such as SOD, CAT, and APX, and their corresponding genes [31, 32], which are partially associated with the discrepancies observed in their regulatory roles in stress response and resistance process. Therefore, the GSH pathway and redox control in relation to water relation as affected by biochar application under drought condition are still in vague.

In the present study, we hypothesized that (1) drought-induced oxidative stress leads to alteration of endogenous hormonal status and GSH synthesis pathway, (2) biochar application improves water relation and alleviates oxidative stress under drought conditions, and (3) specific SA-mediated regulation of GSH synthesis pathway is involved in drought tolerance, particularly redox modulation. To test these hypotheses, the responses of water relation, ROS, GSH metabolism, GSH-based redox status to drought imposition with or without biochar application were interpreted as linked to those of endogenous hormonal level and their signaling genes, especially in GSH-based redox control process.

## Materials and methods

### Material preparation and characteristics

The experimental soil was sourced from agricultural fields at Chonnam National University (Gwangju, Republic of Korea). The soil was classified as a black-brown inceptisol that had not previously been treated with pig slurry. The soil was a sandy loam with a relatively coarse texture, consisting of 9.4% clay, 26.3% silt, and 65.3% sand. Its chemical properties (g kg^−1^) included 0.150 ± 0.01 total N, 0.024 ± 0.003 NH_4_^+^, 0.013 ± 0.001 NO_3_^−^ and 0.255 ± 0.012 P, with a pH (water, 1:5) of 7.45 ± 0.05. Rice husk biochar (RHB) was supplied by Gumok Inc. (Gyeongju, Gyeongbuk, Korea). Table 1 presents the main physicochemical properties of rice husk biochar used in the experiment.

**Table 1:** Chemical properties of selected biochar

Parameters	Value
Total N (%)	0.02
Total P (%)	0.02
Potassium (%)	1.27
Calcium (%)	0.21
Magnesium (%)	0.05
Sodium (%)	0.01
pH (water, 1:10)	8.72

Values represent means of three independent measurements

### Experimental design

Seeds of *Brassica napus* L. (cv. Mosa) were gifted by a colleague who had purchased them from a local supplier in Caen, France. The seeds were germinated in soil trays and grown in a greenhouse under a 16-h light/8-h dark photoperiod, with an average temperature of 27 ℃ during the day and 20 ℃ at night, and relative humidity maintained from 61 to 71%. At the four-leaf stage, seedlings were carefully transplanted into pots measuring 502 mm (L) × 315 mm (W) × 230 mm (H), with one plant per pot. The plants were maintained under the same greenhouse conditions throughout the experiment. Pots were randomly arranged at the beginning of the experiment and repositioned weekly throughout the treatment period. Each pot was filled with 21 kg of sieved soil, either unamended or amended with RHB at a rate of 10% (w/w) based on dry weight. The pig slurry was applied to each pot at a rate equivalent to 200 kg N ha^−^¹. A corresponding amount of chemical fertilizer was added to each pot to adjust the P_2_O_5_:K_2_O application ratio to 150:150 kg ha^−1^. After 7 days, the plants were subjected to three treatments combining soil type and watering regime: well-watered (Control), drought stress (Drought), or drought stress with biochar application (Drought + Biochar). In the well-watered treatment, water was applied to maintain 80% of the field capacity. In the drought stress treatment, irrigation was withheld until the soil moisture dropped to 40% of field capacity, after which this moisture level was maintained throughout the experimental period using a soil moisture meter (WT-1000 H, Mirae E&I, Geumcheon-gu, Seoul, Korea). This 40% field capacity threshold represents moderate drought stress condition as established by D’Oria et al. [33], who defined 40% field capacity as moderate drought and 25% field capacity as severe drought. This condition enabled the assessment of long-term drought effects on the physiological, biochemical, and molecular responses of *Brassica napus*. Each treatment consisted of three independent pots, and each pot was considered a biological replicate. Leaves from the single plant in each pot were harvested, pooled, and used as one sample per replicate for all biochemical and physiological measurements. The samples were immediately frozen in liquid nitrogen and stored − 80 ℃ until further analysis.

### Soil water retention

Prior to the experiment, the soil water content (SWC) corresponding to 100% field capacity was determined for each treatment by weighing soil-filled pots after full saturation and again after drying for 3 days at 80℃. Field capacity values for 20–80% were subsequently calculated using the same method. Concurrently, soil moisture was also measured using a portable soil moisture meter. A regression equation was then established by correlating the gravimetric water content (20–80% field capacity) with the corresponding readings from the portable soil moisture meter. The regression equation was subsequently used to maintain the soil moisture at 80% field capacity for the control treatment and 40% for the drought stress treatment throughout the experimental period. Similar equations were established to relate SWC to readings from the portable soil moisture meter. The soil moisture was measured daily at 08:00 using the portable meter, prior to the onset of significant surface evaporation.

### Physiological parameters in leaves

Relative water content (RWC) was determined following the method described by Kim et al. [34]. Chlorophyll concentration was measured by soaking leaf tissue in dimethyl sulfoxide and recording absorbance at 645 and 663 nm. Concentrations of chlorophyll a and b were calculated using the formula reported by Hiscox and Israelstam [35]. *In situ* visualization of superoxide anion radicals (O₂⁻) and H₂O₂ in leaf discs was performed by staining with 0.1% nitroblue tetrazolium and 0.1% 3,3’-diaminobenzidine solutions, respectively, as previously described [10, 36]. O₂⁻ concentration was quantified using the hydroxylamine oxidation method [37]. The H₂O₂ concentration was determined by its reaction with 0.1% titanium chloride, following a previously described method [38]. Lipid peroxidation was assessed using the thiobarbituric acid assay, as described by Cakmak and Horst [39], which estimates the level of malondialdehyde (MDA) as an indicator of oxidative damage.

### Glutathione biosynthesis

Fresh leaf tissue (200 mg) was homogenized in 5% 5-sulfosalicylic acid and centrifuged at 12,000 × g for 10 min at 4 °C. Glutathione content was determined using a GSH/GSSG microplate assay kit (GT40; Oxford Biomedical Research). GR activity was measured following the procedure outlined by Hasanuzzaman et al. [40]. The enzyme extract was incubated with a reaction mixture containing 100 mM potassium phosphate buffer (pH 7.8), 1 mM EDTA, 1 mM GSSG, and 0.2 mM NADPH. GR activity was quantified by monitoring the decrease in absorbance at 340 nm owing to NADPH oxidation, using an extinction coefficient of ε = 6.2 mM⁻¹ cm⁻¹.

### Phytohormone analysis

Plant hormone extraction was conducted using a modified QuEChERS protocol, as described by Flores et al. [41]. Fresh leaf samples were extracted with 1% acetic acid in acetonitrile, followed by purification with C18 and MgSO₄. The purified supernatant was filtered and analyzed by LC–MS/MS using a Nexera X2 UHPLC system (Shimadzu) coupled with a QTRAP 4500 triple quadrupole-linear ion trap MS (SCIEX), equipped with a reverse-phase C18 column. Analyses were conducted at the High-Tech Materials Analysis Core Facility, Gyeongsang National University (Jinju, Korea). Hormone separation was performed using a water–acetonitrile gradient containing 0.1% formic acid at a flow rate of 0.3 mL min^−1^ at 40 °C.

### RNA extraction and quantitative RT-qPCR analysis

Total RNA was extracted from 100 mg of leaf tissue using RNAiso Plus reagent (Takara, China). First-strand cDNA was synthesized using the GoScript Reverse Transcription Kit (Promega, USA). RT-qPCR was performed using SYBR Premix Ex Taq (Takara) on a Bio-Rad system with gene-specific primers (Supplementary Table S1). Gene expression levels were normalized to actin. Each assay was conducted in duplicate as technical replicates for each of the three biological replicates.

### Statistical analyses

The experiment was conducted using a completely randomized design with three biological replicates per treatment. Data were subjected to one-way ANOVA followed by Duncan’s multiple range test at *P* ≤ 0.05. All analyses were performed using SAS software (version 9.1.3; SAS Institute, Cary, NC, USA). Correlation analysis and principal component analysis (PCA) were conducted using SRplot (http://www.bioinformatics.com.cn/srplot).

## Results

### Soil water status, relative water content, shoot biomass, and chlorophyll concentration

**Fig. 1:**
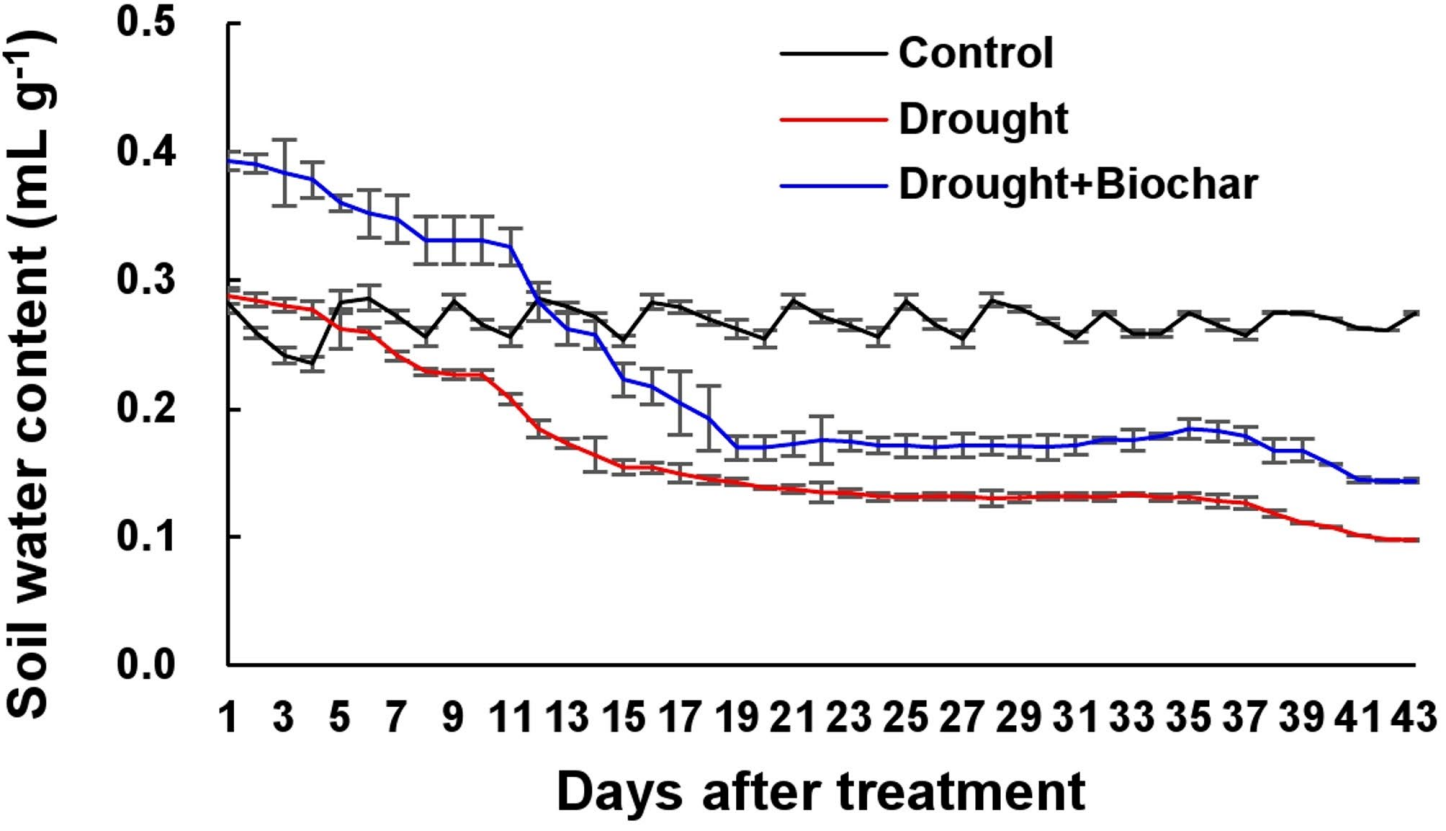
Effect of biochar application on soil water content under drought conditions for 43 days. Treatments include control, drought, and drought with biochar application (Drought + Biochar). Values represent means ± standard error (SE) with n = 3

**Fig. 2:**
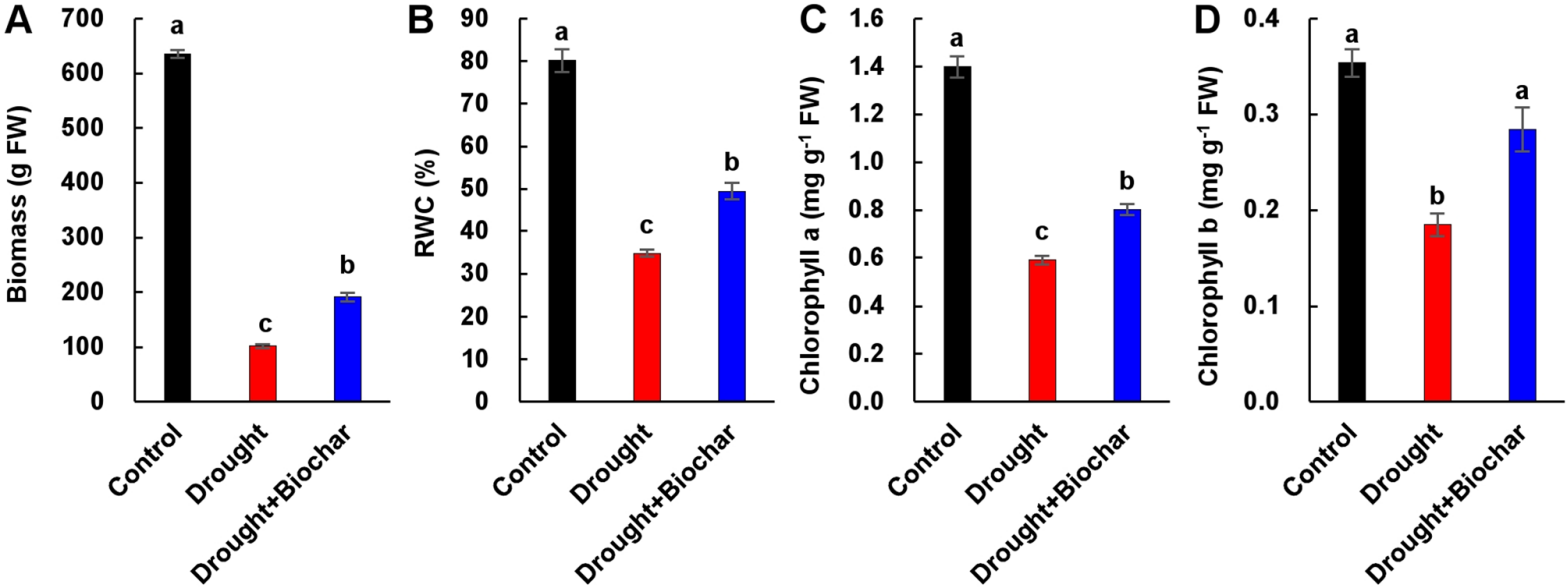
Effect of biochar application on shoot biomass (**A**), relative water content (RWC, **B**), and concentration of chlorophyll a (**C**) and chlorophyll b (**D**) in the leaves of *Brassica napus* under drought conditions for 43 days. Treatments include control, drought, and drought with biochar application (Drought + Biochar). Values represent means ± standard error (SE) with *n* = 3. Different letters indicate statistically significant differences among treatments at *P* ≤ 0.05 according to Duncan’s multiple range test

Drought stress significantly reduced SWC, RWC, and shoot biomass compared to the well-watered control (Figs. 1 and 2). SWC declined steadily until day 17 and then remained relatively stable throughout the experimental period. On day 43, the reduction in SWC was less pronounced in drought stress with biochar application (Drought + Biochar) treatment (48.1% decrease relative to the control) than in the drought alone treatment (64.0% decrease) (Fig. 1). Drought alone reduced shoot biomass and RWC by 84.0% and 56.6%, respectively, compared to the control. However, both parameters were substantially improved in the Drought + Biochar plants (Fig. 2A, B). Drought-induced reduction of chlorophyll a and b concentrations was alleviated by biochar application (Fig. 2C, D).

###  Reactive oxygen species status and lipid peroxidation

**Fig. 3:**
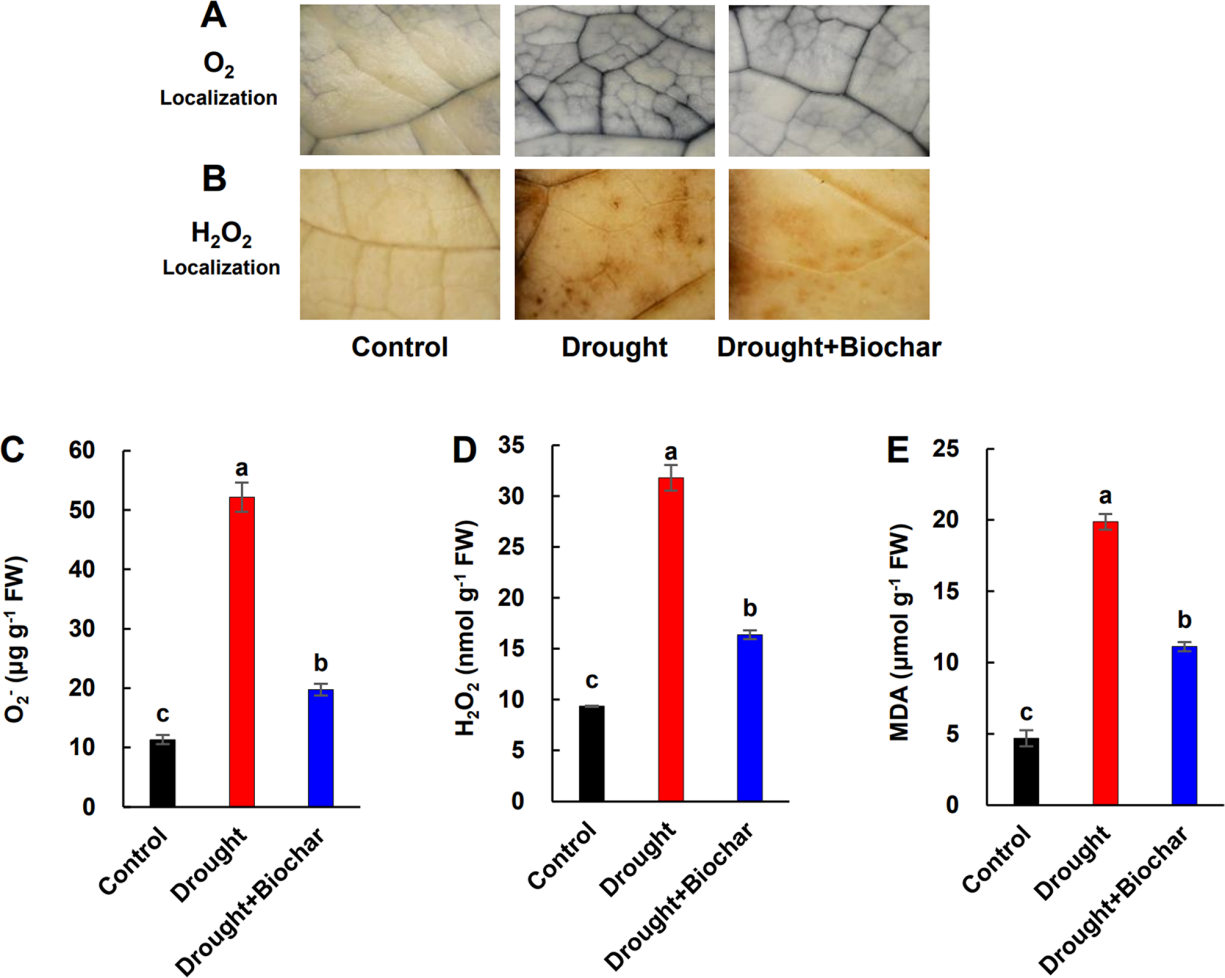
Effect of biochar application on the localization of hydrogen peroxide (H₂O₂, A) and superoxide anion (O₂⁻, B), and the concentration of O₂⁻ (C), H₂O₂ (D), and malondialdehyde (MDA, E) in the leaves of Brassica napus under drought conditions for 43 days. Treatments include control, drought, and drought with biochar application (Drought+Biochar). O₂⁻ accumulation visualized by nitroblue tetrazolium staining, which is indicated by dark parts. H₂O₂ accumulation visualized by 3,3′-diaminobenzidine staining, which is indicated by dark brown spot. Values represent means ± standard error (SE) with n = 3. Different letters indicate statistically significant differences among treatments at P ≤ 0.05 according to Duncan’s multiple range test.

*In situ* staining revealed widespread dark blue and brown coloration, indicative of O₂⁻ and H₂O₂ accumulation, respectively, under drought conditions. In contrast, Drought + Biochar plants exhibited noticeably weaker staining (Fig. 3A, B). Quantitatively, drought stress increased H₂O₂ and O₂⁻ levels by more than 3.4-fold and MDA (an indicator of lipid peroxidation) by 4.2-fold compared to the control. However, Drought + Biochar reduced these increases by 44.0–62.2% compared drought alone (Fig. 3C–E).

### Glutathione metabolism and expression of glutathione biosynthesis genes

**Fig. 4:**
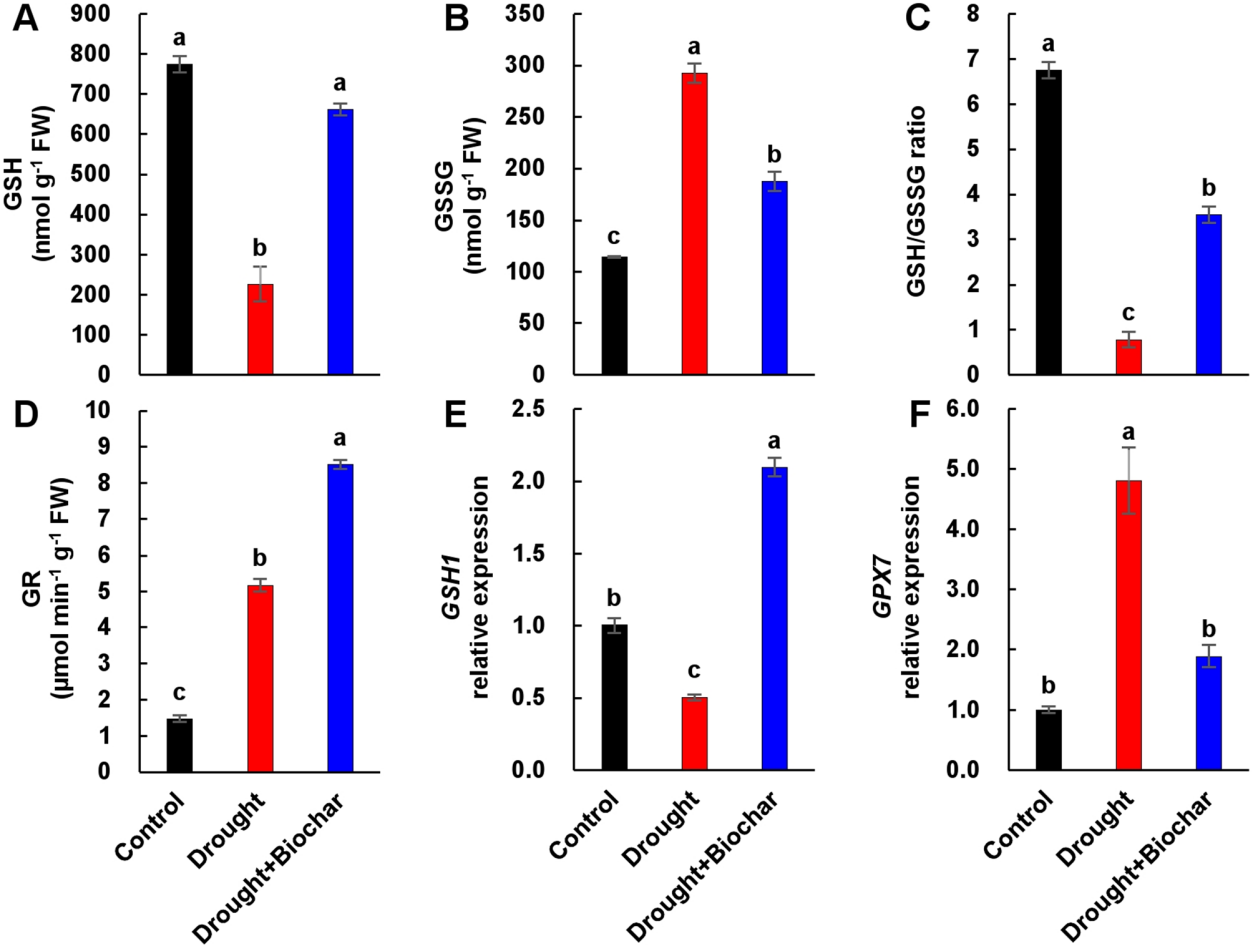
Effect of biochar application on the glutathione pool and related gene expression in the leaves of *Brassica napus* under drought conditions for 43 days: reduced glutathione (GSH, **A**), oxidized GSH (GSSG, **B**), GSH/GSSG ratio (**C**), GSH reductase activity (GR, **D**), and expression of *GSH biosynthesis gene 1* (*GSH1*, **E**) and *glutathione peroxidase 7* (*GPX7*, **F**). Treatments include control, drought, and drought with biochar application (Drought + Biochar). Values represent means ± standard error (SE) with *n* = 3. The expression levels of the control plants were set to 1. Different letters indicate statistically significant differences among treatments at *P* ≤ 0.05 according to Duncan’s multiple range test

Drought stress led to a 70.8% decrease in reduced GSH levels and a 2.6-fold increase in oxidized GSH (GSSG), resulting in an 88.3% reduction in the GSH/GSSG ratio compared to the control (Fig. 4A–C). Biochar treatment significantly increased GSH levels, suppressed GSSG accumulation, and improved the GSH/GSSG ratio by more than 4.5-fold compared to drought alone (Fig. 4A–C). The activity of GR, a key enzyme in GSH recycling, was elevated under drought alone compared to control, and further enhanced by biochar application (Fig. 4D). The expression of *GSH biosynthesis gene 1* (*GSH1*) was strongly downregulated under drought alone but significantly upregulated following biochar treatment (Fig. 4E). In contrast, the expression of *GSH peroxidase 7* (*GPX7*) gene, which encodes GSH peroxidase, was strongly induced 4.8-fold by drought alone compared to the control. However, biochar application under drought conditions attenuated this induction, resulting in only a 1.9-fold increase compared to the control, which represents a 60.7% decrease in expression relative to drought alone (Fig. 4F).

### Hormone levels and the expression of hormone biosynthesis and signaling genes

**Fig. 5:**
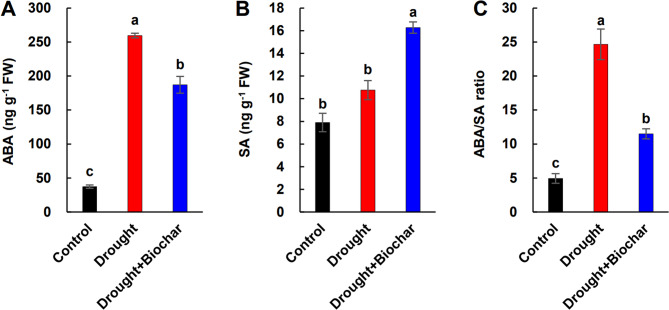
Effect of biochar application on the concentration of abscisic acid (ABA, **A**) and salicylic acid (SA, **B**), and the ABA/SA ratio (**C**) in the leaves of *Brassica napus* under drought conditions for 43 days. Treatments include control, drought, and drought with biochar application (Drought + Biochar). Values represent means ± standard error (SE) with *n* = 3. Different letters indicate statistically significant differences among treatments at *P* ≤ 0.05 according to Duncan’s multiple range test

**Fig. 6:**
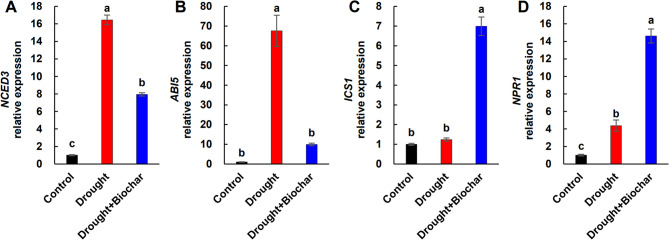
Effect of biochar application on the expression of abscisic acid (ABA) synthesis-related gene *9-cis-epoxycarotenoid dioxygenase 3* (*NCED3*, **A**), ABA signaling-related gene *ABA insensitive 5* (*ABI5*, **B**), salicylic acid (SA) synthesis-related gene *isochorismate synthase 1* (*ICS1*, **C**), and SA signaling-related gene *non-expression of pathogenesis-related gene 1* (*NPR1*, **D**) in leaves of *Brassica napus* under drought conditions for 43 days. Treatments include control, drought, and drought with biochar application (Drought + Biochar). Values represent means ± standard error (SE) with *n* = 3. The expression levels of the control plants were set to 1. Different letters indicate statistically significant differences among treatments at *P* ≤ 0.05 according to Duncan’s multiple range test

ABA levels increased significantly under drought conditions, whereas SA levels showed only a slight increase and were largely similar to those of the control (Fig. 5A, B). The ABA/SA ratio increased more than 5.0-fold under drought alone treatment (Fig. 5C). Biochar treatment significantly enhanced SA content by 51.3% and remarkably reduced the ABA/SA ratio by 53.3% compared to drought alone treatment (Fig. 5A–C). In terms of gene expression, drought stress upregulated *9-cis-epoxycarotenoid dioxygenase 3* (*NCED3*) and *ABA-insensitive 5* (*ABI5*), which are involved in ABA biosynthesis and signaling, respectively (Fig. 6A, B). In contrast, biochar application resulted in a lower induction of both genes compared to drought alone. The SA biosynthesis gene *isochorismate synthase 1* (*ICS1*) was upregulated only in response to biochar treatment, while SA signaling-related gene *non-expression of pathogenesis-related gene 1* (*NPR1*) was upregulated under drought stress and further enhanced by biochar application (Fig. 6C, D).

### Principal component analysis and Pearson’s correlation analysis among metabolites and gene expression profiles

**Fig. 7:**
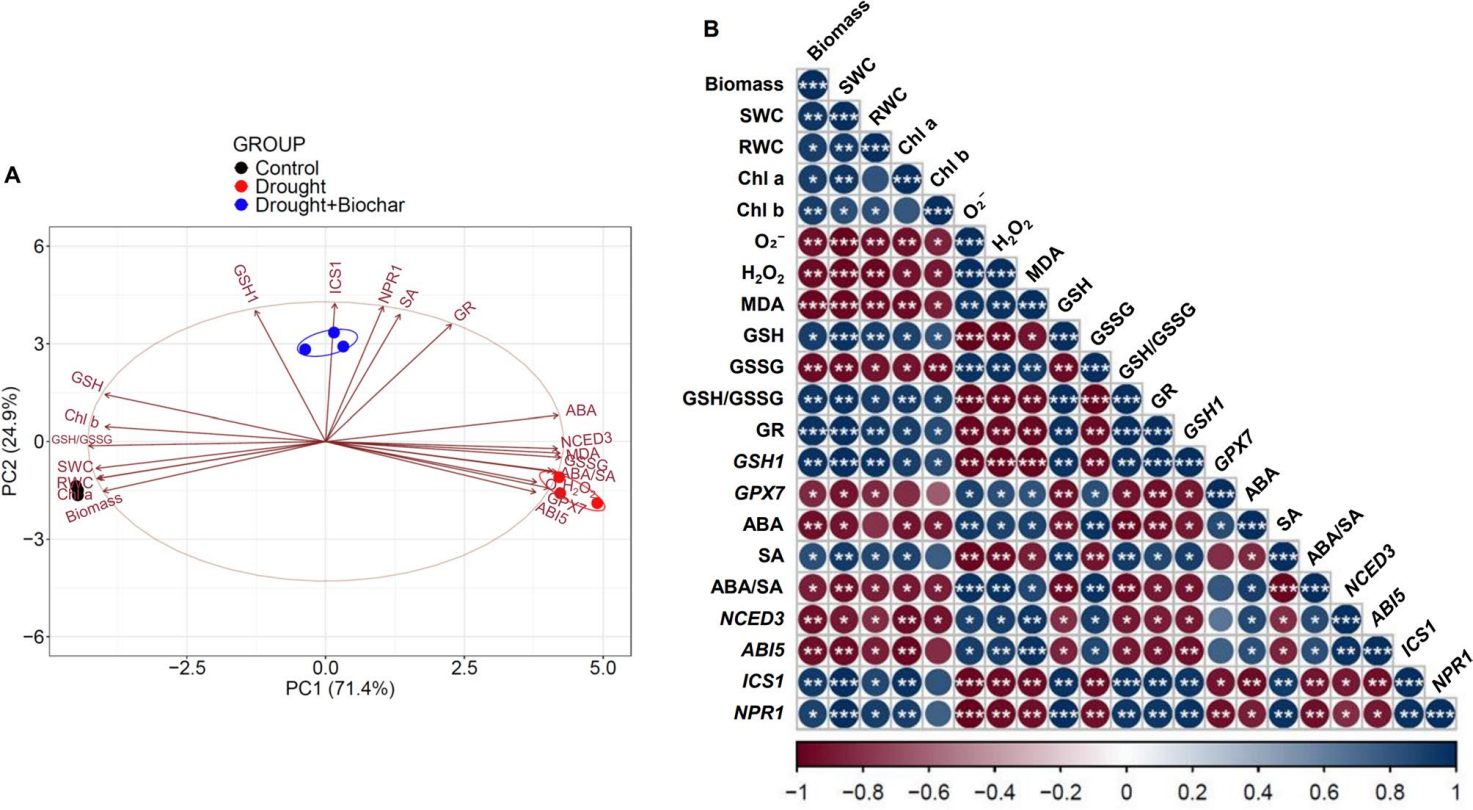
(**A**) Principal component analysis (PCA) biplot illustrating the distribution of physiological, biochemical, and molecular traits of *Brassica napus* under Control (black), Drought (red), and Drought + Biochar (blue) treatments. **B** Pearson’s correlation analysis of physiological and molecular traits under drought and drought with biochar application (Drought + Biochar) conditions. Red and blue indicate negative and positive correlations, respectively, with color intensity proportional to correlation strength. Asterisks indicate levels of statistical significance: ^*^P ≤ 0.05, ^**^P ≤ 0.01, and ^***^P ≤ 0.001. Abbreviations: ABA, abscisic acid; *ABI5*, *ABA insensitive 5*; Chl a, chlorophyll a; Chl b, chlorophyll b; GSH, reduced glutathione; *GSH1*, *GSH biosynthesis gene 1*; *GPX7*, *glutathione peroxidase 7*; GR, glutathione reductase; H₂O₂, hydrogen peroxide; *ICS1*, *isochorismate synthase 1*; MDA, malondialdehyde; *NCED3*, *9-cis-epoxycarotenoid dioxygenase 3*; *NPR1*, *non-expressor of pathogenesis-related gene 1*; O₂⁻, superoxide anion; RWC, relative water content; SA, salicylic acid; SWC, soil water content

Principal component analysis (PCA) was performed to integrate and visualize the overall patterns of physiological and molecular responses under different treatments (Fig. 7A). The first two principal components, PC1 and PC2, accounted for 71.4% and 24.9% of the total variance, respectively, clearly distinguishing the control, drought, and drought with biochar application groups. Drought-associated variables (ABA, *NCED3*, *ABI5*, GSSG, *GPX7*, H_2_O_2_, O₂⁻, MDA) positively loaded on PC1, while biochar-responsive traits (*GSH1*, SA, *NPR1*, *ICS1*, *GR*) showed positive loadings on PC2. To explore the functional relationships between physiological traits, metabolites, and gene expression under drought and Drought + Biochar treatments, Pearson’s correlation coefficients were calculated (Fig. 7B). Shoot biomass and RWC were negatively correlated with ROS and MDA, but positively correlated with reduced GSH and SA. ROS levels were negatively correlated with SA and the ABA/SA ratio. Additionally, GSH showed strong positive correlations with GR and SA-related genes such as *ICS1* and *NPR1*, while showing negative correlations with *GPX7* and ABA-related genes such as *NCED3* and *ABI5*.

## Discussion

### Biochar improves water status and alleviates oxidative stress under drought conditions

Drought stress disrupts plant water balance and induces excessive accumulation of ROS, including O₂⁻ and H₂O₂, leading to oxidative damage to lipids, proteins, and cellular structures [17, 36, 42]. Our results demonstrated that drought stress significantly elevated ROS and MDA contents (Fig. 3), while markedly reducing RWC, chlorophyll levels, and shoot biomass (Fig. 2). These quantitative changes provide direct evidence of severe oxidative and physiological damage in *Brassica napus*. Biochar application significantly alleviated these drought-induced harmful effects by improving water retention and antioxidant capacity. The highly porous structure of biochar, along with its polar surface functional groups, increases the water-holding capacity of the soil [43, 44]. In addition, biochar alters the distribution of soil pore sizes, a key determinant of water retention and storage [20, 45]. Consequently, improved soil moisture availability facilitates root water uptake and helps maintain higher RWC in leaves under drought stress [46]. This enhanced SWC positively influenced plant water status, as reflected by enhanced RWC (Figs. 1 and 2B). The strong positive correlation between SWC and RWC (Fig. 7B) provides evidence that soil hydration improvements directly enhance to physiological stability during drought. This enhanced cellular hydration likely stabilized plasma membrane integrity and reduced oxidative damage, as reflected by lower MDA content and reduced ROS accumulation [47]. Importantly, biochar application significantly decreased O₂⁻ and H₂O₂ levels by 62.2% and 48.5%, respectively, compared to drought alone treatment, accompanied by a marked 44.0% reduction in MDA content (Fig. 3). Similar reductions in oxidative stress have been reported in various crops [32, 47], our findings further demonstrate that biochar-mediated ROS reduction is closely related to improved physiological water status and membrane protection under drought. In addition to its physical effects on water retention, biochar may also trigger biochemical defense responses. Notably, we observed a potential link between decreased oxidative damage and the activation of GSH-related antioxidant pathways (Fig. 7), which are known to play central roles in redox homeostasis under drought conditions [6].

### Biochar-mediated restoration of glutathione redox homeostasis under drought conditions

In its reduced form, GSH not only scavenges ROS directly but also acts as a key electron donor in the AsA–GSH cycle, thereby supporting cellular redox homeostasis. Accordingly, both the concentration of reduced GSH and GSH/GSSG ratio are widely recognized as reliable indicators of intracellular redox status. In this study, drought stress significantly decreased the level of reduced GSH by 70.8%, increased the accumulation of GSSG by 2.6-fold, and sharply decreased the GSH/GSSG ratio by 88.3% (Fig. 4A–C), indicating a severe redox imbalance. The shift toward a more oxidized GSH pool indicates that the cellular antioxidant system was overwhelmed and unable to neutralize the excessive accumulation of ROS [8, 10, 17]. Biochar application effectively reversed these drought-induced disruptions by elevating reduced GSH levels, lowering GSSG accumulation, and restoring the GSH/GSSG ratio (Fig. 4A–C), thereby re-establishing redox homeostasis. While similar improvements in GSH status have been observed under salinity stress [48, 49], our results demonstrate that biochar enhances redox balance in *Brassica napus* under drought conditions by modulating both enzymatic and transcriptional responses. In particular, the biochar-induced the higher increase in GR activity, an enzyme responsible for recycling GSSG into GSH, likely contributed to the enhanced GSH pool along with suppression of *GPX7* expression (Fig. 4). Positive correlation between GR activity and GSH levels (Fig. 7B) further supports this association. The role of GR in maintaining GSH-dependent redox homeostasis has been well documented under drought conditions [7, 50]. In this study, the pronounced upregulation of GR, particularly under biochar treatment, suggests that it functioned as a compensatory mechanism to counteract drought-induced GSSG accumulation.

This redox regulatory mechanism was further supported by gene expression data. Drought stress downregulated *GSH1* expression, a key gene in GSH biosynthesis, while upregulating *GPX7*, which encodes GSH peroxidase responsible for GSH oxidation. In contrast, biochar treatment reversed these effects by upregulating *GSH1* and downregulating *GPX7* expression (Fig. 4E, F), thereby promoting GSH synthesis and reducing its consumption. This observation is consistent with previous findings involving the ROS scavenger dimethylthiourea, where increased GR activity and reduced *GPX7* expression were associated with improved redox balance [17]. Furthermore, reduced *GPX7* expression has been associated with lower H₂O₂ accumulation in *Arabidopsis chloroplasts* [51]. In this study, the elevation of GR activity and reduced *GPX7* expression under biochar coincide with reduced ROS and higher growth performance (Fig. 7B). These findings suggest that biochar-mediated *GPX7* suppression may directly mitigate oxidative stress at the subcellular level. Taken together, these results provide strong evidence that biochar enhances the non-enzymatic antioxidant system by simultaneously promoting GSH biosynthesis, recycling, and limiting its oxidation. This integrated transcriptional and enzymatic response reinforces ROS detoxification capacity and restores cellular redox homeostasis under drought stress.

### Salicylic acid-mediated redox regulation is key to biochar-induced drought tolerance

Beyond enhancing the GSH redox system, our findings indicate that hormonal regulation—particularly the antagonistic interaction between SA and ABA—plays a critical role in modulating GSH metabolism under drought stress. Drought conditions led to a slight increase in SA content but suppressed the expression of SA-responsive genes (*ICS1* and *NPR1*), whereas the ABA levels increased markedly, accompanied by the upregulation of ABA biosynthesis and signaling genes (*NCED3* and *ABI5*) (Figs. 5 and 6), consistent with ABA-dominant stress responses previously reported [16, 17, 52]. This hormonal shift corresponds with heightened oxidative stress and disrupted redox homeostasis, as evidenced by reduced levels of decreased GSH, elevated accumulation of GSSG, and a lowered GSH/GSSG ratio (Fig. 4A-C). This redox imbalance appears to be aggravated by ABA-mediated upregulation of *GPX7* despite increased GR activity, indicating insufficient GSH recycling capacity under high ABA conditions (Fig. 4D, F). Our results support earlier findings that ABA enhances *GPX* expression and GSSG levels, promoting H₂O₂ accumulation [16, 18]. A positive correlation between ABA levels and *GPX7* expression, along with negative correlation between ABA and GSH/GSSG ratio (Fig. 7B), reinforce the inhibitory role of ABA on redox balance under drought stress. By contrast, SA is known to enhance antioxidant capacity by activating enzymes such as CAT, DHAR, APX, and GR, thereby promoting the scavenging of H_2_O_2_ and stimulating GSH biosynthesis [10, 11, 53]. Our previous study demonstrated that elevated SA signaling under mild drought was associated with reduced ROS accumulation and suppression of ABA-related responses [16]. Additionally, SA-deficient *Arabidopsis* mutants (e.g., *sid2*, *cat2sid2*) exhibit reduced GSH levels, whereas SA-overproducing lines accumulate higher GSH [51, 54, 55], highlighting the critical function of SA in redox regulation.

In the present study, biochar application effectively mitigated drought-induced redox and hormonal imbalances by modulating SA–ABA crosstalk. Biochar-treated plants exhibited elevated SA levels and upregulation of SA-responsive genes (*ICS1* and *NPR1*), while simultaneously suppressing ABA accumulation and the expression of ABA-related genes (*NCED3* and *ABI5*) (Figs. 5 and 6). This hormonal rebalancing was closely associated with the restoration of GSH homeostasis, as reflected by increased GSH content, elevated GSH/GSSG ratio, and enhanced GR activity (Fig. 4). These findings support established evidence that SA positively regulates GSH biosynthesis by inducing the transcription of *GSH1* and *GR* [53, 56]. Furthermore, exogenous SA treatment mitigates ABA accumulation while increasing GSH levels and improving the GSH/GSSG ratio [10, 15, 54]. These outcomes confirm that SA represses ABA biosynthesis and signaling [52, 57], while ABA promotes *GPX* expression and GSSG content during oxidative stress [16]. Furthermore, biochar application significantly downregulated *GPX7* expression alongside a reduction in ABA levels (Figs. 4F and 5A), indicating a potential antagonistic interaction between SA and ABA in regulating GSH-based redox homeostasis. PCA analysis further supported that ABA-related stress markers (e.g., *GPX7*, GSSG, and ROS indicators) were primarily associated with drought responses, while biochar treatment clustered with SA-related genes (*ICS1*, *NPR1*), GR, and *GSH1*, highlighting a distinct SA-GSH regulatory signature (Fig. 7A). Pearson’s correlation analysis also revealed strong positive correlations among SA levels, GSH content, GR activity, and the expression of SA-regulated genes (Fig. 7B). Collectively, these findings indicate that biochar enhances drought tolerance by activating the SA-GSH interaction, thereby restoring redox balance and suppressing ABA-mediated oxidative responses.

## Conclusion

Biochar-amended soil maintained higher soil moisture, which contributed to increased RWC, preservation of chlorophyll pigments, and sustained shoot biomass under drought stress. In consequence, biochar application mitigated drought-induced GSH oxidation by elevating reduced GSH, lowering GSSG, thereby restoring the GSH-based redox balance. These redox improvements were associated with increased GR activity, upregulation of the GSH‐biosynthetic gene *GSH1*, and suppression of *GPX7*. The protective effect of biochar application was also closely linked to shift in hormonal balance. Whereas drought alone produced an ABA-dominant profile associated with GSH depletion and oxidative damage, biochar application elevated SA levels with an antagonistic suppression of ABA accumulation with upregulated SA-responsive genes *ICS1* and *NPR1*, leading to an elevated GSH/GSSG ratio. Taken together, it thus concludes that biochar application mitigates drought-induced oxidative stress by integrating improved water relation with redox regulation in the SA-mediated GSH synthesis pathway. In our best knowledge, the present study firstly characterized the physiological significance of biochar application under drought condition in modulating phytohormone-mediated GSH-based redox control as part of drought tolerance mechanism in *Brassica napus*. Thus, rice husk biochar application may have important implication in an agricultural perspective for improving drought stress tolerance. Furthermore, metabolic engineering techniques based on SA-mediated GSH-redox control revealed by biochar application will contribute to increase the sustainability and productivity of field crops under drought stress. Future field studies across diverse soil types and different water regimes are necessary to validate the biochar-mediated drought tolerance mechanism identified in the present study.

## Supplementary Information

Supplementary Material 1.

## Data Availability

The data provided in this study are available from the corresponding author upon reasonable request.
