# Supplementary material for: Biochar application alleviates drought-induced oxidative stress by activating the salicylic acid-mediated glutathione synthesis pathway in Brassica napus
Source: BMC Plant Biol. 2025 Nov 17;25:1582. doi: 10.1186/s12870-025-07575-7 (PMC12625075; doi:10.1186/s12870-025-07575-7)
Supplement: Supplementary file 1 — Supplementary Material 1. [file 12870_2025_7575_MOESM1_ESM.docx]

**Supplementary Table S1** Specific primers used for qRT-PCR

| Genes | Forward primer (5’–3’) | Reverse primer (5’–3’) |
| --- | --- | --- |
| *BnGSH1* | TGAATCAGGGGAAACAAAGC | TGACTTCAGCACAGGTTTGG |
| *BnGPX7* | GGATATCAAGCGGTTCGTGT | GTCGTGGGAGGGTATCTCTG |
| *BnNCED3* | GGAGTGCTTCTGCTTCCATC | TTCGAGGTTGACTTGCTCCT |
| *BnABI5* | AACAAACACCTGGCTGAACC | TGAGGATTTCCTGGTGAAGG |
| *BnICS1* | TCAATCCCAGAACGAGATCC | GACAGAAACCTTCGGATGGA |
| *BnNPR1* | TGAGAACATTGCCAAGCAAG | CAACAGCAAAATGGAGAGCA |
| *BnActin7* | GATTCCGTTGCCCTGAAGTA | GCGACCACCTTGATCTTCAT |
